# Influences on pre-hospital delay in the diagnosis of colorectal cancer: a systematic review

**DOI:** 10.1038/sj.bjc.6604096

**Published:** 2007-12-04

**Authors:** E Mitchell, S Macdonald, N C Campbell, D Weller, U Macleod

**Affiliations:** 1 School of Health and Social Care, Glasgow Caledonian University, Cowcaddens Road, Glasgow G4 0BA, UK; 2 Division of Community Based Sciences, General Practice and Primary Care, University of Glasgow, 1 Horselethill Road, Glasgow G12 9LX, UK; 3 General Practice and Primary Care, University of Aberdeen, Foresterhill Health Centre, Westburn Road, Aberdeen AB25 2AY, UK; 4 Community Health Sciences (General Practice), University of Edinburgh, 20 West Richmond Street, Edinburgh EH8 9DX, UK

**Keywords:** colorectal, delay, diagnosis, systematic review

## Abstract

Colorectal cancer is a major global health problem, with survival varying according to stage at diagnosis. Delayed diagnosis can result from patient, practitioner or hospital delay. This paper reports the results of a review of the factors influencing pre-hospital delay – the time between a patient first noticing a cancer symptom and presenting to primary care or between first presentation and referral to secondary care. A systematic methodology was applied, including extensive searches of the literature published from 1970 to 2003, systematic data extraction, quality assessment and narrative data synthesis. Fifty-four studies were included. Patients' non-recognition of symptom seriousness increased delay, as did symptom denial. Patient delay was greater for rectal than colon cancers and the presence of more serious symptoms, such as pain, reduced delay. There appears to be no relationship between delay and patients' age, sex or socioeconomic status. Initial misdiagnosis, inadequate examination and inaccurate investigations increased practitioner delay. Use of referral guidelines may reduce delay, although evidence is currently limited. No intervention studies were identified. If delayed diagnosis is to be reduced, there must be increased recognition of the significance of symptoms among patients, and development and evaluation of interventions that are designed to ensure appropriate diagnosis and examination by practitioners.

Colorectal cancer is a major global health problem and the fourth most common cause of cancer death worldwide (Parkin *et al*, 2001). It is also a cancer the incidence of which continues to grow, particularly in developed countries (Parkin *et al*, 1999). Survival varies according to stage at diagnosis with 5-year survival falling from almost 90% for early cancers (Dukes A) to 15% for advanced tumours, when only palliative resection is possible (McArdle and Hole, 2002). In the majority of cases, primary care is the first point of contact for patients with lower gastrointestinal cancer symptoms, and colorectal cancer is not always an immediate diagnosis. Associated symptoms, including rectal bleeding and altered bowel habit, are common in primary care practice and as such, general practitioners (GPs) are required to differentiate between patients whose symptoms may be due to cancer and the much larger number of patients whose symptoms are attributable to benign, self-limiting illness.

The complexity surrounding identification of those patients requiring further investigation has led to the production of guidelines in many countries, all with the aim of encouraging earlier diagnosis (Benson *et al*, 2000; Department of Health, 2000; National Institute for Health and Clinical Excellence, 2005; Australian Cancer Network, 2006). In addition, patients too must determine whether a symptom warrants presentation to the health service or requires adoption of a ‘wait and see approach’. As such, delayed diagnosis of colorectal cancer can occur as a result of patient delay (the time between first noticing a symptom and first consulting a doctor), practitioner delay (between first consultation and referral) or hospital delay (between referral and diagnosis) (Nichols *et al*, 1981) and greater knowledge of the factors contributing to these phases is required if survival is to be increased. We conducted a systematic review of the factors that influence pre-hospital delay, that is, up to the point of referral.

## MATERIALS AND METHODS

A worldwide review of the literature from 1970 to 2003 was conducted to identify the reasons for delay by patients in presentation with cancer symptoms and by practitioners in cancer detection and referral. Studies were identified from electronic databases (Medline, EMBASE, CINAHL, PsycINFO, Science Citation Index, Social Science Citation Index, International Bibliography of the Social Sciences, Proceedings First and Web of Science Proceedings), Cochrane Collaboration review groups, bibliographies, books, citations in identified articles and authors active in the field. Studies were selected if they focused on adult cancer and (1) the participants were individuals or groups of patients or primary care practitioners and (2) they evaluated factors associated with the interval between a patient first noticing a cancer symptom and presenting to primary care, or described an intervention designed to reduce that interval or (3) they evaluated factors associated with the time interval between the patient first presenting to primary care and being referred to secondary care, or described an intervention designed to reduce that interval. To identify factors associated with help-seeking and referral behaviour, studies that determined patient attitudes towards cancer awareness and presentation were also included, as were those which determined provider attitudes or behaviour towards cancer referral. Studies evaluating delay from presentation to treatment were not excluded until they were reviewed to ensure that they did not differentiate between stages in the delay cycle. Studies assessing the outcome of delay in terms of diagnosis, treatment or patient outcomes were excluded, as were those considering only the cost of interventions, validity of referral decisions or differences in referral periods.

Following the initial search, all references were independently assessed, and if subsequently eligible for inclusion, rated by two reviewers (SM, UM). Where differences of opinion occurred, papers were validated by a third reviewer (EM) and findings discussed until a consensus was reached. Previously developed scoring systems were extended and applied to assess the methodological adequacy of studies (Mitchell and Sullivan, 2001). Many used methodologies that did not lend themselves to such techniques; therefore, each included study was also assessed on the strength of the evidence it presented. We determined three grades of evidence: strong, moderate and insufficient.

Studies providing strong evidence were those with an adequate sample size, rigorous methods to ascertain data (i.e. not open to selection bias) and reporting statistically significant differences in relation to the delay-related factors identified (or using appropriate analytic techniques if qualitative). Studies providing moderate evidence had an adequate sample size, reported significant differences but used less rigorous methods to ascertain data or had an adequate sample size, used rigorous methods to ascertain data but used comparative analysis or reported only relevant descriptive statistics, without statistical testing of differences. Studies providing insufficient evidence had unclear or inappropriate methods to ascertain data and insufficient analysis. Where a study inferred results, the strength of its evidence was downgraded. The full methods used in this review have been described in detail elsewhere (Macdonald *et al*, 2006). Narrative synthesis of findings was undertaken to identify key concepts and themes that were shared across individual studies.

## RESULTS

**Figure 1:**
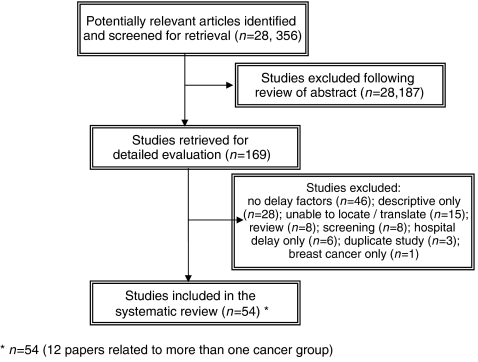
Flow of studies into the review.

The search strategy identified 28 356 articles of which only 169 (0.6%) met the inclusion criteria and were subject to detailed review (Figure 1). Fifty-four papers were included in the final analysis. Cohen's kappa was used to determine inter-rater reliability, that is, the level of concurrence between the two independent reviewers in relation to whether identified studies were considered eligible for detailed evaluation. Kappa co-efficient for agreement beyond chance was 0.52.

More than half of the included studies (*n*=31) were carried out in Western Europe, over half of these in the UK. None employed a controlled trial methodology, with most involving review of medical records or structured interviews with patients. More than one-third investigated both patient and practitioner delay (*n*=20), almost half studied patient factors only (*n*=25) and the remainder studied practitioner factors only. Studies most commonly evaluated any colorectal cancer, with smaller numbers dealing specifically with cancer of the rectum (11%), colon (9%) or anus (4%). Twenty-six papers were assessed as providing strong evidence, 19 provided moderate evidence and 9 provided insufficient evidence.

Studies were comparatively small in size, involving between 17 and 2525 participants (mean 420; median 228). The period under study ranged from 3 months to 53 years. Although more than half of the studies considered practitioner-related delay factors, only five included primary care practitioners as subjects. In almost three-quarters of studies, participants were identified from secondary care (*n*=38), and were either in-patients (39%), outpatient attendees (16%), a combination of the two (5%) or identified from hospital records (37%). Other sources used were cancer registries, census or other household directories and patient groups. Only 6 of the 54 studies recruited patients from primary care.

### Delay intervals

Thirty-eight studies reported length of delay, either from patient recognition of symptoms to presentation (*n*=36) or from presentation to practitioner referral (*n*=24). This was reported in a non-standardised way, and less than half reported intervals in medians, despite delay times having typically skewed distributions. The most frequently used methods of deriving delay intervals were by structured patient interview or data abstraction from hospital records. Only five studies used primary care records (9%). Median patient delay ranged from 7 days to 5 months (Worden and Weisman, 1975; Turunen and Peltokallio, 1982; MacArthur and Smith, 1984; Funch, 1985; Robinson *et al*, 1986; Ratcliffe *et al*, 1989; Dent *et al*, 1990; Curless *et al*, 1994; Arbman *et al*, 1996; Porta *et al*, 1996; Mulcahy and O'Donoghue, 1997; Majumdar *et al*, 1999; Mariscal *et al*, 2001) and practitioner delay from 0 to 15 months (Turunen and Peltokallio, 1982; MacArthur and Smith, 1984; Funch, 1985; Ratcliffe *et al*, 1989; Mansson, 1990; Jones and Dudgeon, 1992; Curless *et al*, 1994; Arbman *et al*, 1996; Harris and Simson, 1998; Majumdar *et al*, 1999; Mariscal *et al*, 2001).

### Factors influencing patient delay

**Figure 2:**
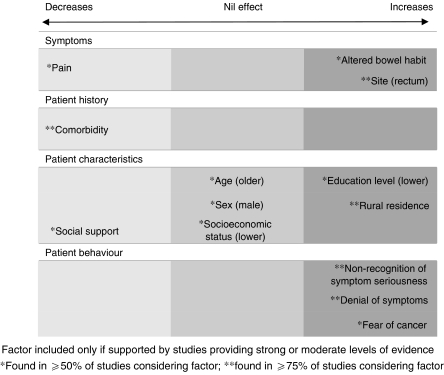
Main factors associated with patient delay and direction of influence.

Forty-four papers considered factors that influenced patient delay. Most (*n*=41) identified factors that increased delay, whereas almost two-thirds (*n*=27) identified factors that decreased delay (Figure 2).

### Patient behaviour

The influence of symptom awareness, and more particularly patients' interpretation of symptoms, was a common theme across studies. Non-recognition of the seriousness of symptoms (Worden and Weisman, 1975; Holliday and Hardcastle, 1979; Rubin *et al*, 1980; Anon, 1982; Marshall and Funch, 1986; Mor *et al*, 1990; Prohaska *et al*, 1990; Byles *et al*, 1992; Curless *et al*, 1994; Porta *et al*, 1996; Roncoroni *et al*, 1999; Sladden *et al*, 1999; Young *et al*, 2000; de Nooijer *et al*, 2001; Cockburn *et al*, 2003), or lack of knowledge, either about the disease itself or about the availability of screening was a major contributor to increased delay (Anon, 1982, 1986; Camilleri-Brennan and Steele, 1999; Pullyblank *et al*, 2002; McCaffery *et al*, 2003). Patients who presented late tended to either deny their symptoms entirely, or redefine these in relation to benign disease (Worden and Weisman, 1975; Bain *et al*, 2002; Langenbach *et al*, 2003). Perhaps unsurprisingly, increased delay was also found for patients who were less worried about their symptoms (Dent *et al*, 1990) or who self-diagnosed or self-medicated before presenting to primary care (Funch, 1985; Dent *et al*, 1990; Tanum *et al*, 1991; Byles *et al*, 1992; Sladden *et al*, 1999; Cockburn *et al*, 2003) (Table 1).

The anxiety associated with recognising a potential cancer symptom was also a key factor in the decision to present. Fear that symptoms were indicative of cancer (Prohaska *et al*, 1990; Byles *et al*, 1992; de Nooijer *et al*, 2001), fear of investigations related to diagnosis of cancer (Langenbach *et al*, 2003) and fear of powerlessness (Worden and Weisman, 1975; McCaffery *et al*, 2003) made patients consult less quickly, although, for some, fear that a symptom might be a sign of cancer brought about more rapid presentation (Hackett *et al*, 1973; Dent *et al*, 1990; Sladden *et al*, 1999; de Nooijer *et al*, 2001).

### Presenting symptom and patient history

For the most part, patients who suffered from more serious symptoms such as obstruction or abdominal pain delayed less (Devlin *et al*, 1973; MacAdam, 1979; Rubin *et al*, 1980; MacArthur and Smith, 1984; Prohaska *et al*, 1990; Mulcahy and O'Donoghue, 1997; Majumdar *et al*, 1999; Young *et al*, 2000; Mariscal *et al*, 2001), whereas those experiencing either nonspecific symptoms or more common symptoms, such as bleeding or altered bowel habit, delayed longer (Devlin *et al*, 1973; Galloway *et al*, 1984; Mor *et al*, 1990; Curless *et al*, 1994). There were, however, some patients for whom pain resulted in increased delay (Hackett *et al*, 1973; Nilsson *et al*, 1982; MacArthur and Smith, 1984; Prohaska *et al*, 1990). Those who recognised the symptom or who had previous experience of a symptom or of cancer itself tended to delay less (MacDonald and Freeling, 1986; Samet *et al*, 1988; Dent *et al*, 1990; Porta *et al*, 1996; Sladden *et al*, 1999). This was also the case for those with comorbidity (Ratcliffe *et al*, 1989; Mor *et al*, 1990; Porta *et al*, 1996; Mariscal *et al*, 2001) and those experiencing multiple symptoms (Mariscal *et al*, 2001).

A number of studies considered the relationship between presentation behaviour and cancer site. These demonstrated that those with cancer of the rectum were more likely to have delayed than patients with colon cancer (Hackett *et al*, 1973; Ratcliffe *et al*, 1989; Arbman *et al*, 1996; Mulcahy and O'Donoghue, 1997; Harris and Simson, 1998). In addition, there was some evidence to suggest that patients with colon cancer may delay less than patients with other cancers such as melanoma (de Nooijer *et al*, 2001).

### Patient characteristics

Social networks and support were identified as being a potentially important factor in reducing delay, when patients either sought advice from or made decisions based on the experience of others (Holliday and Hardcastle, 1979; MacAdam, 1979; MacArthur and Smith, 1984; Samet *et al*, 1988; Camilleri-Brennan and Steele, 1999; Roncoroni *et al*, 1999; Sladden *et al*, 1999). By and large, patient age (Worden and Weisman, 1975; MacAdam, 1979; McDermott *et al*, 1981; Turunen and Peltokallio, 1982; Pitluk and Poticha, 1983; Galloway *et al*, 1984; MacArthur and Smith, 1984; Marshall and Funch, 1986; Robinson *et al*, 1986; Samet *et al*, 1988; Dent *et al*, 1990; Mor *et al*, 1990; Prohaska *et al*, 1990; Marble *et al*, 1992; Kemppainen *et al*, 1993; Curless *et al*, 1994; Arbman *et al*, 1996; Porta *et al*, 1996; Mulcahy and O'Donoghue, 1997; Majumdar *et al*, 1999; Mariscal *et al*, 2001; McCaffery *et al*, 2003) and sex (Worden and Weisman, 1975; MacAdam, 1979; McDermott *et al*, 1981; Turunen and Peltokallio, 1982; Marshall and Funch, 1986; Robinson *et al*, 1986; Samet *et al*, 1988; Dent *et al*, 1990; Kemppainen *et al*, 1993; Porta *et al*, 1996; Mulcahy and O'Donoghue, 1997; Majumdar *et al*, 1999; Sladden *et al*, 1999; Young *et al*, 2000; Mariscal *et al*, 2001; Cockburn *et al*, 2003; McCaffery *et al*, 2003) had no impact on delay. Furthermore, there was no relationship between delay and lower socioeconomic status (Hackett *et al*, 1973; Worden and Weisman, 1975; MacAdam, 1979; MacArthur and Smith, 1984; Samet *et al*, 1988; Prohaska *et al*, 1990; Porta *et al*, 1996; Langenbach *et al*, 2003), although some studies suggested that this might be associated with increased delay. Rural residence (Robinson *et al*, 1986; Bain *et al*, 2002) and lower levels of education (Marshall and Funch, 1986; Dent *et al*, 1990; Vineis *et al*, 1993; Porta *et al*, 1996; Mariscal *et al*, 2001; Cockburn *et al*, 2003; McCaffery *et al*, 2003) were both found to increase delay.

### Factors influencing practitioner delay

**Figure 3:**
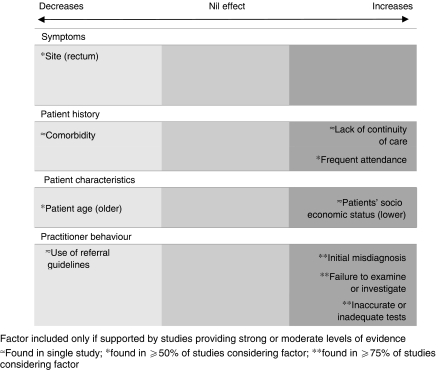
Main factors associated with practitioner delay and direction of influence.

Twenty-nine papers considered factors that influence practitioner delay. More than three-quarters (*n*=24) identified factors that increased delay and less than half (*n*=12) factors that decreased delay (Figure 3).

### Practitioner behaviour

The most commonly identified themes associated with delayed referral related to initial diagnosis and activity of the practitioner. Misdiagnosis, occurring either through treating patients symptomatically or attributing symptoms to a health problem other than colorectal cancer, resulted in increased time to referral (Spasov, 1978; Holliday and Hardcastle, 1979; Rubin *et al*, 1980; Zaichuk, 1980; Nilsson *et al*, 1982; Turunen and Peltokallio, 1982; Funch, 1985; Dixon *et al*, 1990; Mansson, 1990; Edwards *et al*, 1991; Harris and Simson, 1998; Roncoroni *et al*, 1999; Young *et al*, 2000). In addition, failure to examine the patient, usually rectal examination (Spasov, 1978; Holliday and Hardcastle, 1979; Rubin *et al*, 1980; Zaichuk, 1980; Turunen and Peltokallio, 1982; MacArthur and Smith, 1984; Dixon *et al*, 1990; Mansson, 1990; Tanum *et al*, 1991; Kemppainen *et al*, 1993; Roncoroni *et al*, 1999; Young *et al*, 2000; Langenbach *et al*, 2003), or receiving negative or false negative test results (Funch, 1985; Kemppainen *et al*, 1993; Harris and Simson, 1998) contributed to the delay. One qualitative study suggested that early presentation on the part of the patient could actually increase delay if disease went undetected or was misdiagnosed as benign (Bain *et al*, 2002). In addition, some patients identified practitioners as gatekeepers and a potential barrier to their referral since the patient waited for the GP to act on their behalf (Bain *et al*, 2002). Although limited, there is some evidence to suggest that appropriate referral and use of referral guidelines is associated with reduced delay (Holliday and Hardcastle, 1979; Debnath *et al*, 2002; Eccersley *et al*, 2003). Practitioners in rural areas were less likely to refer, due to the distance from specialist services (Sladden and Thomson, 1998) (Table 2).

### Presenting symptom

Although the nature of symptoms will undoubtedly have contributed to referral decisions, it was difficult to reach definitive conclusions about their influence. For some patients, experiencing pain resulted in more rapid referral (Mariscal *et al*, 2001), whereas for others this had no impact (Young *et al*, 2000). Similarly, presenting with rectal bleeding could lead both to a more rapid (Sladden and Thomson, 1998; Mariscal *et al*, 2001) or more delayed outcome (Rubin *et al*, 1980; Mansson, 1990; Edwards *et al*, 1991). Evaluation of the impact of tumour site on referral decision demonstrated that patients with rectal cancers were less likely to experience delay than those with colon cancers (MacAdam, 1979; Marshall and Funch, 1986; Robinson *et al*, 1986; Ratcliffe *et al*, 1989; Arbman *et al*, 1996).

### Patient history

Consultation patterns related to obtaining a diagnosis were also found to be of relevance, with those patients, often women, who frequently consulted their general practitioner following a non-conclusive initial visit, more likely to experience delayed referral (MacAdam, 1979; Turunen and Peltokallio, 1982; Marshall and Funch, 1986). Similarly, those lacking continuity of care could also suffer delay, although a second opinion might precipitate referral (Bain *et al*, 2002). Patients with co-existing disease were likely to be referred more quickly (Mariscal *et al*, 2001).

### Patient characteristics

There was some evidence relating to the impact of certain patient characteristics on practitioners' referral patterns. Older patients were in general referred more quickly (Nilsson *et al*, 1982; Turunen and Peltokallio, 1982; Pitluk and Poticha, 1983; Robinson *et al*, 1986; Sladden and Thomson, 1998), and there is some evidence of an association between delay and social class, with those from the lower end of the socioeconomic spectrum experiencing a longer wait to referral (MacArthur and Smith, 1984). There was however, no conclusive relationship between patient sex and referral delay (Turunen and Peltokallio, 1982; Marshall and Funch, 1986; Robinson *et al*, 1986; Arbman *et al*, 1996).

## DISCUSSION

The importance of colorectal cancer in terms of its burden to society is well established; the benefits of presentation and diagnosis early in the course of the disease are clear. Yet although early diagnosis is desirable, it is also difficult and delays can occur at various points in the process. Understanding why delay occurs is the first step to reducing it. This paper presents a comprehensive, systematic review of the literature relating to the reasons for patient and primary care delay in the diagnosis of colorectal cancer. We found evidence of an association between delay and an extensive number of factors concentrated around four emergent themes: symptoms, patient history, patient characteristics and behaviour.

A strength of this review is the inclusion of studies in any language, reducing the potential for bias introduced by the exclusion of papers published in non-English language journals, which may be more inclined to show negative results (Egger *et al*, 1997). The main limitation of the review relates to substantial heterogeneity between included studies. The nature of the topic and variability in study quality and reporting made it neither possible nor appropriate to pool data for meta-analysis. Rather, we graded study evidence by the robustness of its methodology and analysis, allowing us to weight each study in our composite assessment of delay-related factors. A previous systematic review of delay in breast cancer concluded that most studies were of poor quality and that the strength of evidence was inadequate to inform development of strategies to shorten delay (Ramirez *et al*, 1999). Half of all studies included in this review provided strong evidence in relation to the factors they reported, and a further third provided moderate evidence. It is encouraging to note that most of these papers have been published since 1990; almost half in the last decade. Furthermore, we identified considerably more evidence than previous narrative and systematic reviews conducted as part of evidence-based guidelines (Carter and Winslet, 1998; Scottish Intercollegiate Guidelines Network, 2003).

One important finding of this review centres on the complex relationship between presentation behaviour and presenting symptoms. The evidence suggests that if delay is to be reduced, what is important is not simply patients' awareness of symptoms but rather their recognition and understanding of the potential seriousness of those symptoms. The implications of this are not without difficulty. For the majority of patients presenting to primary care, symptoms such as rectal bleeding and change in bowel habit are attributable to benign, self-limiting illness. Interpretation of these symptoms as benign will therefore usually be correct. However, for the minority of patients whose symptoms are due to colorectal cancer, delays if long enough may lead to more advanced stage disease and less chance of cure. Thus, considerable emphasis must be placed on highlighting the potentially significant nature of symptoms, despite their commonality. The challenge lies in achieving a suitable balance, which targets the appropriate population without creating undue fear, overburdening primary care services with patients seeking reassurance or clogging up scarce investigative services. This is particularly important given the paradoxical relationship that can exist between delay and fear of a potential cancer symptom.

Although some patients denied their symptoms or re-defined them in relation to benign disease, self-diagnosis and self-treatment were common themes across studies. This may go some way to explaining why patient delay was found to be greater for rectal than for colon cancers. It is likely that many people will associate rectal bleeding with haemorrhoids or some other benign ano-rectal problem. Embarrassment may deter presentation. As such, patients may not attend with the symptom until it becomes problematic; thus for example, the presence of pain reduces delay. Interestingly, patients with comorbidity also delayed less, perhaps due to their already frequent attendance in practice and the ease with which new problems could then be discussed.

The most common reasons for practitioner delay related to initial misdiagnosis and insufficient examination. This is in keeping with findings from previous reviews of delay in cancer diagnosis and is similar to those reported for hospital-related delay (Goodman and Irvin, 1993; Potter and Wilson, 1999). Lower gastrointestinal symptoms are common in patients presenting to primary care and the challenge of appropriate referral is a significant one. The complexity surrounding identification of those patients requiring further investigation has led to the production of several guidelines, all with the aim of facilitating earlier diagnosis. Implicit within these is examination, either to determine whether the patient has an abdominal or rectal mass, or to confirm the existence of a benign explanation. Yet, this review suggests that at least a quarter of patients and perhaps as many as three quarters do not receive a rectal examination (Holliday and Hardcastle, 1979; Rubin *et al*, 1980; Nilsson *et al*, 1982; Turunen and Peltokallio, 1982; MacArthur and Smith, 1984; Dixon *et al*, 1990; Kemppainen *et al*, 1993; Roncoroni *et al*, 1999; Young *et al*, 2000; Langenbach *et al*, 2003). The full impact of the use of referral guidelines is not yet clear; there is some evidence to suggest that they may reduce delay, but the strength of that evidence to date is limited. Furthermore, we found no intervention studies related to reducing patient or practitioner delay for colorectal cancer. Consequently, the impact of existing initiatives, such as guidelines, must be investigated further.

The NHS is currently rolling out a bowel cancer screening programme; it commenced in England and is due to achieve nationwide coverage by 2009 (http://cancerscreening.org.uk/bowel). The programme targets men and women aged 60–69 (50–74 in Scotland) and offers biennial screening via home faecal occult blood test kits. The full impact of the programme on patients' response to bowel symptoms is likely to be complex and will require evaluation. Nonetheless, it is likely that such screening will have some influence on pre-hospital delay, possibly through raising awareness of bowel symptoms and their potential seriousness, with consequent earlier presentation.

However, the bowel screening programme is aimed at detecting early stage disease in asymptomatic patients. Consequently, delays caused by some factors identified in this review, such as fear of cancer, denial of symptoms, initial practitioner misdiagnosis or failure to fully examine patients with rectal bleeding, will most likely be unaffected by the screening programme. Furthermore, a negative screening result may give patients false reassurance if they subsequently develop symptoms, an occurrence that has already been found to contribute to practitioner delay (Funch, 1985; Kemppainen *et al*, 1993; Harris and Simson, 1998).

The findings from this review would suggest that the way ahead, although clear, is also complex. If we are to reduce delay in the pre-hospital phase of colorectal cancer diagnosis, we must address two main areas. Firstly, we must overcome the dilemma faced by patients, that of when to categorise nonspecific symptoms as non-serious. Attributing symptoms to benign disease may be entirely appropriate and as such, difficult to influence. Achieving this may include public education, but it is also likely to involve greater awareness of how symptoms are interpreted in the context of pre-existing disease, patient experience, social circumstances and life priorities. Such focus, away from ensuring recognition of symptoms and towards improving understanding of symptoms, will in turn require a shift in thinking on the part of the medical and research communities.

Secondly, we must influence the circumstances under which practitioners decide that further examination, investigation and referral are appropriate. This may require changes to existing guidelines, whereby physical examination and laboratory investigation are made explicit rather than implicit in the decision to refer. It may also require a change in practice, resulting in physical examination of all those who present with lower gastrointestinal symptoms, regardless of age, previous history or symptom duration. The initiation of such changes may already be underway, and indeed the recently published NICE Referral Guidelines for Suspected Cancer, which this review was intended to inform, now explicitly state that digital rectal examination should always be carried out in patients with unexplained lower GI symptoms (National Institute for Health and Clinical Excellence, 2005). What is certain is that pre-hospital delay in colorectal cancer is avoidable and it must be addressed if outcomes and survival are to be improved.

## Figures and Tables

**Table 1:** Patient associated delay factors

**Author(s)**	**Location**	**Study type**	**Participants**	**Cancer site**	**Factors which increase delay**	**Factors which decrease delay**	**No impact on delay**	**Evidence**
Devlin *et al* (1973)	England	Retrospective observational	310 patients (aged 30–95, 53% men, 47% women)	Rectum	Symptom type – altered bowel habit, bleeding	Symptom type – abdominal or ano-rectal pain		Moderate
Hackett *et al* (1973)	Massachusetts, USA	Prospective observational	563 patients (aged 17–91, mean 62; 46% men, 54% women); 17% with colon/rectal cancer)	Colon, rectum	Symptom type – pain; cancer site – rectum; social class – lower; procrastination; worry over health; family history	Worry; incapacitated by symptoms; acknowledgment of cancer		Strong
Worden and Weisman (1975)	Massachusetts, USA	Prospective observational	125 patients (aged 19–59, 38% men, 62% women), 22% with colon cancer	Colon	Non-recognition of symptom seriousness; denial; powerlessness; comorbidity; fatigue		Age; sex; marital status; socioeconomic status; family history	Strong
Holliday and Hardcastle (1979)	England	Prospective observational	200 patients (58% men, mean age 66; 42% women mean age 67)	Colorectal	Non-recognition of symptom seriousness	Advice from social network		Moderate
MacAdam (1979)	England	Prospective observational	150 patients (79% with colon/rectal cancer), 105 GPs	Colon, rectum	Cancer site – rectum	Cancer site – colon; symptom type – abdominal pain, bleeding	Socioeconomic status; age; sex; social isolation; frequency of consulting	Moderate
Rubin *et al* (1980)	Israel	Prospective observational	100 patients (aged 36–85, mean 64; 66% men, 34% women)	Colorectal	Non-recognition of symptom seriousness	Symptom type – abdominal pain, weakness		Strong
McDermott *et al* (1981)	Australia	Retrospective observational	1228 patients (55% men, mean age 61; 45% women, mean age 59)	Rectum			Age; sex; symptom type	Moderate
Anon (1982)	USA	Cross-sectional	804 members of the public (aged 40+)	Colorectal	Non-recognition of symptom seriousness; lack of knowledge; lack of routine screening^a^			Insufficient
Nilsson *et al* (1982)	Sweden	Retrospective observational	284 patients (aged 20–99; 52% men, 48% women)	Colorectal	Symptom type – pain, bleeding, bowel disturbance^a^			Insufficient
Turunen and Peltokallio (1982)	Finland	Prospective observational	100 patients (45% men, 55% women	Colorectal	Age – <50; sex – male			Moderate
Pitluk and Poticha (1983)	Illinois, USA	Retrospective observational	826 patients (31 aged ⩽40; 45% men, 55% women)	Colorectal	Age – younger			Moderate
Galloway *et al* (1984)	Scotland	Retrospective observational	481 patients (92.5% aged 50+; 50% men, 50% women)	Colorectal	Age – younger; symptom type – rectal bleeding			Strong
MacArthur and Smith (1984)	England	Prospective observational	127 patients	Colorectal	Symptom type – weight loss, rectal pain;	Symptom type – abdominal pain, nausea; advice from social network	Age; social class	Strong
Funch (1985)	Washington, USA	Prospective observational	294 patients (aged 18–85; 49% men, 51% women)	Colorectal	Other life events; feeling better; self-treatment			Moderate
Anon (1986)	USA	Cross-sectional	2525 members of the public (aged 40+)	Colorectal	Lack of awareness of screening; lack of knowledge^a^			Insufficient
MacDonald and Freeling (1986)	England	Cross-sectional	171 GP patients (aged 55+; 50% men, 50% women)	Colorectal		Recognition of symptoms; symptom type – bleeding^a^		Moderate
Marshall and Funch (1986)	Washington, USA	Prospective observational	306 patients (aged 18–85; 50% men, 50% women)	Colorectal	Sex – female; cancer site – rectum (female)/colon (male); non-recognition of symptom seriousness		Age; education	Strong
Robinson *et al* (1986)	Israel	Retrospective observational	445 patients (54% men, 46% women)	Colorectal	Widowhood	Residence – urban	Age; sex	Strong
Samet *et al* (1988)	New Mexico, USA	Prospective observational	800 patients (aged 65–100, mean 72), 28% with colorectal cancer	Colorectal	Race – white Hispanic; sex – male; income – lower	Previous cancer diagnosis; regular check-ups	Age; availability of vehicle; social support; participation in screening	Strong
Ratcliffe *et al* (1989)	England	Prospective observational	332 patients (aged 30–100, mean 70; 51% men, 49% women)	Colorectal	Family history; cancer site – rectum	Comorbidity – diverticular disease		Strong
Dent *et al* (1990)	Australia	Cross-sectional	93 patients with rectal bleeding (aged 35–85, median 55; 54% men, 46% women), 58 GPs	Colorectal	Consulting non-medical professional; self-treatment; less worry (self-diagnosis); education level – lower	Previous rectal bleeding; regularly checking toilet paper or faeces; worry that bleeding means cancer	Age; sex; social support; income; ethnicity; occupation	Strong
Mor *et al* (1990)	Rhode Island, USA	Prospective observational	625 patients (aged 45–90; 31% men, 69% women), 46% with colorectal cancer	Colorectal	Non-recognition of symptom seriousness; age – younger; symptom type – bleeding, altered bowel habit	Comorbidity		Strong
Prohaska *et al* (1990)	Washington, USA	Prospective observational	254 patients (48% men; 52% women)	Colon, rectum	Non-recognition of symptom seriousness; symptom type – rectal pain; too busy; fear	Symptom type – abdominal pain	Age; income	Strong
Tanum *et al* (1991)	Norway	Retrospective observational	117 patients (aged 35–91; 21% men, 79% women)	Anus	Self-treatment			Insufficient
Byles *et al* (1992)	Australia	Cross-sectional	1221 members of public (aged 40+; 49% men; 51% women), 20% with rectal bleeding	Rectum	Non-recognition of symptom seriousness; embarrassment; fear; self-diagnosis			Moderate
Marble *et al* (1992)	Connecticut, USA	Retrospective observational	100 patients (50 aged 14–40, mean 36; 50 aged 49–86, mean 70)	Colorectal	Age – younger			Strong
Kemppainen *et al* (1993)	Finland	Retrospective observational	178 patients (aged 27–97, mean 91; 44% men, 56% women)	Colorectal	Age and sex – male <65, female 80+			Moderate
Vineis *et al* (1993)	Italy	Prospective observational	330 patients, 29% with colon cancer (58% men, 42% women)	Colon		Education level – higher		Strong
Curless *et al* (1994)	England	Prospective observational	273 patients (aged 25–93, median 68; 56% men, 44% women)	Colorectal	Presentation with non-specific symptoms; non-recognition of symptom seriousness		Age	Strong
Arbman *et al* (1996)	Sweden	Retrospective observational	554 patients (aged 30–95; 51% men, mean age 70; 49% women, mean age 72), 39% with rectal, 61% with colon cancer	Colon, rectum	Cancer site – rectum;	Presenting as emergency	Age	Strong
Porta *et al* (1996)	Spain	Prospective observational	183 patients (mean age 67; 66% men, 34% women)	Colon, rectum	Age – older; sex – male; illiteracy; social class – lower; unemployment; non-recognition of symptom seriousness	Age – younger; comorbidity; recognition of symptom seriousness	Marital status; family history	Strong
Mulcahy and O'Donoghue (1997)	Ireland	Prospective observational	777 patients (aged 26–92, mean 68; 54% men, 46% women)	Colorectal	Age – younger; cancer site – rectum	Symptom type – obstruction	Sex	Strong
Camilleri- Brennan and Steele (1999)	Scotland	Cross-sectional	1004 adult members of the public (mean age 50, 40% men, 60% women)	Colorectal	Lack of knowledge^a^	Experience through social network^a^		Moderate
Majumdar *et al* (1999)	North Carolina, USA	Retrospective observational	194 patients (aged 15–95, mean 66; 53% men, 47% women)	Colorectal	Symptom type – weight loss	Symptom type – obstruction	Age; sex; cancer site	Strong
Roncoroni *et al* (1999)	Italy	Prospective observational	100 patients (aged 38–89; 54% men, 46% women)	Colorectal	Non-recognition of symptom seriousness	Advice from social network		Strong
Sladden *et al* (1999)	Australia	Cross-sectional	903 GP attenders (aged 50+, mean 66; 44% men, 56% women)	Rectum	Non-recognition of symptom seriousness; self-treatment; previous rectal bleeding; sex – male	Blood in toilet; advice from social network; worry that bleeding means cancer		Strong
Young *et al* (2000)	Australia	Prospective observational	100 patients (aged 43–92, mean 70; 52% men, 48% women)	Colorectal	Sex – male; non-recognition of symptom seriousness	Symptom type – pain, bleeding		Strong
de Nooijer *et al* (2001)	The Netherlands	Qualitative interviews	23 patients (mean age 52; 43% men, 57% women), 26% with colon cancer, 10 GPs	Colon	Non-recognition of symptom seriousness; cancer-site – colon; fear of cancer	Fear of cancer; trust in GP		Strong
Mariscal *et al* (2001)	Spain	Prospective observational	217 patients (aged 59–74, mean 65; 59% men, 41% women), 73% with colon cancer	Large bowel	Education level – higher	Comorbidity; symptom type – pain, bleeding; first presenting at hospital; multiple symptoms	Age; sex; availability of vehicle	Strong
Bain *et al* (2002)	Scotland	Qualitative interviews	61 patients, 34 relatives	Colorectal	Symptom denial or re-definition; early presentation; residence – rural			Strong
Pullyblank *et al* (2002)	England	Cross-sectional	77 GP attenders (aged 19–77, median 42; 40% men, 60% women)	Colorectal	Lack of awareness^a^	Improved media publicity^a^		Moderate
Cockburn *et al* (2003)	Australia	Cross-sectional	1332 members of public (aged 40+; 40% men; 60% women)	Colorectal	Non-recognition of symptom seriousness; self-diagnosis; sex – female; marital status – married^a^	Education level – higher; higher perception of risk; belief in benefit of early detection^a^		Moderate
Langenbach *et al* (2003)	Germany	Prospective observational	70 patients (54% men, mean age 68, 46% women, mean age 65), 57% with colon and 43% with rectal cancer	Colon, rectum	Fear of investigation; symptom denial; marital status – divorced; income – welfare	Additional health insurance; marital status – married		Moderate
McCaffery *et al* (2003)	UK wide	Cross-sectional	1637 members of public (aged 16–74; 46% men, 54% women)	Colorectal	Lack of awareness; negative attitude about cancer^a^		Knowledge; age; sex; education level^a^	Moderate

a Study infers finding.

**Table 2:** Practitioner associated delay factors

**Author(s)**	**Location**	**Study type**	**Participants**	**Cancer site**	**Factors which increase delay**	**Factors which decrease delay**	**No impact on delay**	**Evidence**
Spasov (1978)	Russia	Retrospective observational	382 patients	Rectum	Initial misdiagnosis; inadequate investigation			Unable to determine
Holliday and Hardcastle (1979)	England	Prospective observational	200 patients (58% men, mean age 66; 42% women mean age 67)	Colorectal	Failure to examine; initial misdiagnosis; inappropriate referral			Moderate
MacAdam (1979)	England	Prospective observational	150 patients (79% with colon/rectal cancer), 105 GPs	Colon, rectum	Cancer site – colon	Cancer site – rectum	Regular consulting rate of patient	Moderate
Rubin *et al* 1980)	Israel	Prospective observational	100 patients (aged 36–85, mean 64; 66% men, 34% women)	Colorectal	Initial misdiagnosis; failure to examine; symptom type – bleeding			Strong
Zaichuk (1980)	Russia	Retrospective observational	55 patients	Rectum	Failure to examine; initial misdiagnosis			Unable to determine
Nilsson *et al* (1982)	Sweden	Retrospective observational	284 patients (aged 20–99; 52% men, 48% women)	Colorectal		Adequate examination; Accurate tests^a^	Patient age	Insufficient
Turunen and Peltokallio (1982)	Finland	Prospective observational	100 patients (45% men, 55% women	Colorectal	Patient age – <50; patient sex – male; initial misdiagnosis; failure to examine; frequent attendance by patient			Moderate
Pitluk and Poticha (1983)	Illinois, USA	Retrospective observational	826 patients (31 aged ⩽40; 45% men, 55% women)	Colorectal	Patient age – younger			Moderate
MacArthur and Smith (1984)	England	Prospective observational	127 patients	Colorectal	Failure to examine; patient social class – lower	Symptom type – constipation		Strong
Funch (1985)	Washington, USA	Prospective observational	294 patients (aged 18–85; 49% men, 51% women)	Colorectal	Initial misdiagnosis; inaccurate tests			Moderate
Marshall and Funch (1986)	Washington, USA	Prospective observational	306 patients (aged 18–85; 50% men, 50% women)	Colorectal	Patient sex – female; cancer site – colon; frequent attendance by patient			Strong
Robinson *et al* (1986)	Israel	Retrospective observational	445 patients (54% men, 46% women)	Colorectal	Cancer site – rectum		Patient age; patient sex	Strong
Ratcliffe *et al* (1989)	England	Prospective observational	332 patients (aged 30–100, mean 70; 51% men, 49% women)	Colorectal	Cancer site – rectum	Cancer site – left sided carcinoma		Strong
Dixon *et al* (1990)	England	Retrospective observational	376 patients (aged 31–91, median 67) referred by 151 GPs	Colorectal	Initial misdiagnosis; failure to examine^a^	Awareness^a^		Insufficient
Mansson (1990)	Sweden	Retrospective observational	42 patients (aged 45–92; 43% men, 57% women)	Colorectal	Initial misdiagnosis; failure to examine; symptom type - bleeding			Moderate
Edwards *et al* (1991)	Wales	Retrospective observational	22 patients (aged 45–81, mean 63; 50% men, 50% women)	Anus	Symptom type – bleeding; initial misdiagnosis			Moderate
Tanum *et al* (1991)	Norway	Retrospective observational	117 patients (aged 35–91; 21% men, 79% women)	Anus	Failure to examine^a^			Insufficient
Jones and Dudgeon (1992)	England	Retrospective observational	245 GPs, 1465 patients (>300 with colon cancer)	Colon		Cancer site – colon		Moderate
Kemppainen *et al* (1993)	Finland	Retrospective observational	178 patients (aged 27–97, mean 91; 44% men, 56% women)	Colorectal	Failure to examine; inaccurate tests			Moderate
Arbman *et al* (1996)	Sweden	Retrospective observational	554 patients (aged 30–95; 51% men, mean age 70; 49% women, mean age 72), 39% with rectal and 61% with colon cancer	Colon, rectum	Patient sex – female	Cancer site – rectum		Strong
Harris and Simson (1998)	England	Retrospective observational	17 patients (aged 43–86, mean 72; 59% men, 41% women)	Colorectal	Initial misdiagnosis; inaccurate tests			Moderate
Sladden and Thomson (1998)	Australia	Cross-sectional	68 GPs (aged 32–67; median 44)	Rectum	Practice location – rural	Patient age – older; symptom type – blood in toilet; no visible cause; multiple episodes of bleeding		Strong
Roncoroni *et al* (1999)	Italy	Prospective observational	100 patients (aged 38–89; 54% men, 46% women)	Colorectal	Initial misdiagnosis; failure to examine			Strong
Young *et al* (2000)	Australia	Prospective observational	100 patients (aged 43–92, mean 70; 52% men, 48% women)	Colorectal	Initial misdiagnosis; failure to examine; misinterpretation of results		Presenting symptom	Strong
Mariscal *et al* (2001)	Spain	Prospective observational	217 patients (aged 59–74, mean 65; 59% men, 41% women), 73% with colon cancer	Large bowel		Comorbidity; symptom type – pain, bleeding		Strong
Bain *et al* (2002)	Scotland	Qualitative interviews	61 patients, 34 relatives	Colorectal	Lack of continuity; undetected disease; gate-keeping			Strong
Debnath *et al* (2002)	England	Retrospective observational	239 referrals; 92 GPs	Colorectal		Compliance with referral guideline^a^		Insufficient
Eccersley *et al* (2003)	England	Prospective observational	180 urgent referrals	Colorectal		Appropriate use of urgent referrals^a^		Insufficient
Langenbach *et al* (2003)	Germany	Prospective observational	70 patients (54% men, mean age 68, 46% women mean age 65), 57% with colon, 43% with rectal cancer	Colon, rectum	Patient marital status – married; failure to examine	Patient marital status – widowed; additional health insurance		Moderate

a Study infers finding; (non-English language paper).
